# Mechanical Respond and Failure Mode of Large Size Honeycomb Sandwiched Composites under In-Plane Shear Load

**DOI:** 10.3390/molecules24234248

**Published:** 2019-11-21

**Authors:** Mo-Nan Wang, Baoqin Wang, Changxi Liu, Guangxin Zhang, Yumin Wan, Fa Zhang

**Affiliations:** 1 School of Mechanical and Power Engineering, Harbin University of Science and Technology, Harbin 150080, China; wangbqhljit@126.com; 2 College of Mechanical and Electronic Engineering, Heilongjiang Institute of Technology, Harbin 150030, China; 13144511966@126.com; 3 Beijing Key Laboratory of Civil Aircraft Structures and Composite Materials, Beijing Aeronautical Science & Technology Research Institute of COMAC, Beijing 102211, China; zhangguangxin@comac.cc; 4 School of Material Science and Engineering, Beihang University, Beijing 100191, China; wanyumin@buaa.edu.cn

**Keywords:** honeycomb sandwiched composite, large size, in-plane shear, mechanical respond, theoretical and FEM analysis, FEM

## Abstract

The present work focuses on the in-plane shear respond and failure mode of large size honeycomb sandwich composites which consist of plain weave carbon fabric laminate skins and aramid paper core. A special size specimen based on a typical element of aircraft fuselage was designed and manufactured. A modified in-plane shear test method and the corresponding fixture was developed. Three large size specimens were tested. The distributed strain gauges were used to monitor the mechanical response and ultimate bearing capacity. The results show that a linear respond of displacement and strain appears with the increase of the load. The average shear failure load reaches 205.68 kN with the shear failure occurring on the face sheet, and the maximum shear strain monitored on the composite plate is up to 16,115 με. A combination of theoretical analysis and finite element method (FEM) was conducted to predict the shear field distribution and the overall buckling load. The out-of-plane displacement field distribution and in-plane shear strain field distribution under the pure shear loading were revealed. The theoretical analysis method was deduced to obtain the variation rule of the shear buckling load. A good agreement was achieved among the experiment, theoretical analysis, and FEM results. It can be concluded that the theoretical analysis method is relatively conservative, and the FEM is more accurate in case of deformation and strain. The results predicted by h element and p element methods are very close. The results of the study could provide data support for the comprehensive promotion of the design and application of honeycomb sandwich composites.

## 1. Introduction

Honeycomb sandwich composite structure is a special type of composite material composed of two stiff face sheets bonded to a very lightweight honeycomb core [1]. The face sheets could be a natural or synthetic fiber reinforced composite [2,3,4,5] or metal [1,6] and the core could be replaced as polymer foam [4,5] or aluminum foam [1,6,7]. This provides the possibility for the engineer to widely change the parameters to meet the design specifications. Due to its high specific stiffness and strength, excellent energy absorption capacity, good noise resistance, as well as high damping properties, the sandwich composite structure has been widely used in the aerospace field [1,2,3,4,5,6,7,8,9,10,11]. The mechanical properties of the sandwich composite structure are highly anisotropic with more failure modes, and the processing technology and structural characteristics of composite materials are more complex than traditional materials, especially alloy materials [12,13,14,15,16]. Therefore, it is of great significance to thoroughly study the mechanical properties of sandwich composite structures for aircraft design [17,18,19,20].

There is an increasing interest in the application of the honeycomb sandwich panels for the body of aircraft [21]. Regarding the aircraft body, the main function of the inner and outer skin is to carry the normal stress due to air pressure and the in-plane shear stress coming from the aircraft wing, while the core is used to connect the two skins and carries the vertical force. This structure can not only play the excellent tensile properties of composite skins, but also enhance stability without redundant structures comparing the traditional stiffened panel [7,22]. Shear force is two forces of equal size and opposite direction in two parallel planes which are very close to each other [23]. The test and evaluation of the in-plane shear strength have great significance for the rational design of the sandwich composite structures, the assurance of their safety, and the expansion of their application scope [24,25,26]. 

Most structural materials have size effect especially for the large size sandwich structure subjected to in-plane shear loading. One of the main difficulties in measuring its shear properties is generating a pure shear stress state in a relatively large field [27]. Moreover, the shear test is the most difficult of all single stress tests for composite materials [28]. In the case of measuring both shear modulus and shear strength output reproducible values on small specimen, the V-notched test method (ASTM standard D7078 or D5379) [27,29] and the in-plane shear test method (ISO 14129) [30] are the best practices in the commonly used methods. 

A summary of early investigations related to the shear test of the sandwich composites based on the ASTM standard test methods experimentally and numerically with small and easily fabricated specimens is presented in [1,31,32,33,34,35]. Laurent [1] provided a comprehensive study on the shear stresses in honeycomb sandwich plates with analytical solution, finite element method (FEM), and experimental verification. Fan et al. [36] simulated panels of different layers and core thickness using a finite element analysis program and made three-point bending tests and shear tests. The results indicated that core thickness played an important role in the panels buckling and post-buckling responses, and the number of carbon fiber layers decided the shear strength. Kim et al. [37] studied the stability of Al honeycomb core sandwiched composite panels via finite element analysis. Wang et al. [38] designed a 3D spacer fabric structure using glass fibers and carbon fibers as raw materials and analyzed the compression and flexure properties of the 3D spacer fabric. Prakash et al. [39] investigated the influence of cell size on the core shear modulus and shear strength of fiber-reinforced plastic (FRP) honeycomb core sandwiched panels. Kolanu [30] focused on the damage assessment of carbon fiber reinforced polymer (CFRP) panels subjected to in-plane shear loading and undergoing large deformation. 

However, investigation on the shear mechanical response of the large scale honeycomb sandwich composites was inadequate. Zhang [40] conducted an investigation on in-plane shear behavior of large-size composite plates with multi-bolt joints. Kolanu [30] proposed a unified approach comprising of digital image correlation (DIC), acoustic emission (AE), strain gages, and linear variable differential transformer (LVDT) for capturing the post-buckling deformations associated with the large scale laminated composite panels with holes under shear loading. Further, Li [41] carried out experimental and numerical investigations on CFRP laminate with a large elliptical size cut-out subjected to shear loading. Therefore, it is necessary to study the shear properties and failure behavior of the large scale honeycomb sandwich composites as they are more in line with engineering structures. 

Taking the aforementioned studies’ analyses, a large size sandwich structure and a corresponding shear fixture were designed. A combination of theoretical analysis and FEM model was conducted to predict the shear field distribution and the overall buckling load. The FEM is based on the h element and p element. Then the mechanical respond and failure mode of the structure under in-plane shear load were revealed by conducting experiments. The theoretical analysis was deduced and its result further verified consistency with the numerical and experimental results. 

## 2. Material and Specimens

The honeycomb sandwich composite panel was produced with a nominal thickness of 20 mm. The thickness of the top and bottom skins was 0.654 mm, and the middle core was 18.69 mm. The materials and manufacturing details are described below.

### 2.1. CFRP Skin and Honeycomb Core

The CFRP skin is made from 3 plies of T700 carbon fiber plain woven fabric [2] reinforced epoxy resin (supplied by AVIC Composite Materials Ltd, Beijing, China) using the hand layup and vacuum bagging technique. The thickness of cured lamina was 0.218 mm with a density of 200 g/m^2^. The stacking sequence of 45°/0°/0° was designed to provide strength and stiffness to the panel. The honeycomb core was made of AC-KH-48 type Nomex with a density of 48 kg/m^3^ manufactured by ARAMICORE, and designed to carry the out-plane shear forces. The single and double wall thicknesses of the Nomex paper were 0.063 mm and 0.126 mm. The distance of the opposite side of the honeycomb was about 4 mm. It is defined that the honeycomb height (3 direction) is the main bearing direction. The mechanical property of the CFRP skin and Nomex honeycomb core are presented in Table 1 [42].

### 2.2. Manufacturing Process

**Figure 1:**
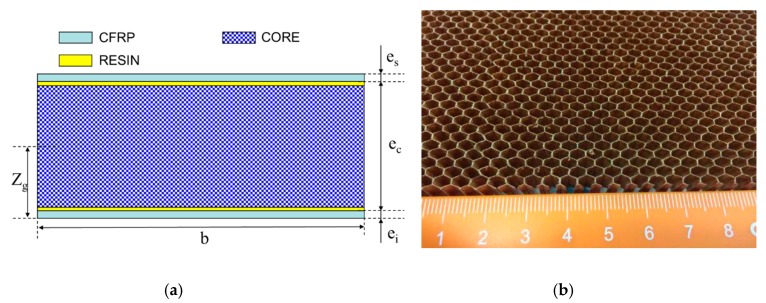
Schematic diagram of Nomex honeycomb sandwich CFRP structure and honeycomb core: (**a**) Nomex honeycomb sandwich CFRP structure; (**b**) honeycomb core (unit: mm).

The Nomex honeycomb sandwich CFRP panels used in this study were manufactured by using a heated press process, in which the panels were cured for 60 min with a pressure of 0.3 MPa at 90 °C. In order to keep the smoothness of the skin surface, the CFRP skins were first cured. Then the CFRP skins and the Nomex honeycomb core were cured together using a toughened structural resin film, EA9696.080K provided by the HENKEL corporation. The schematic diagrams of the honeycomb sandwich structure and the honeycomb core are shown in Figure 1. 

### 2.3. Dimension Design of Sandwich Panel 

**Figure 2:**
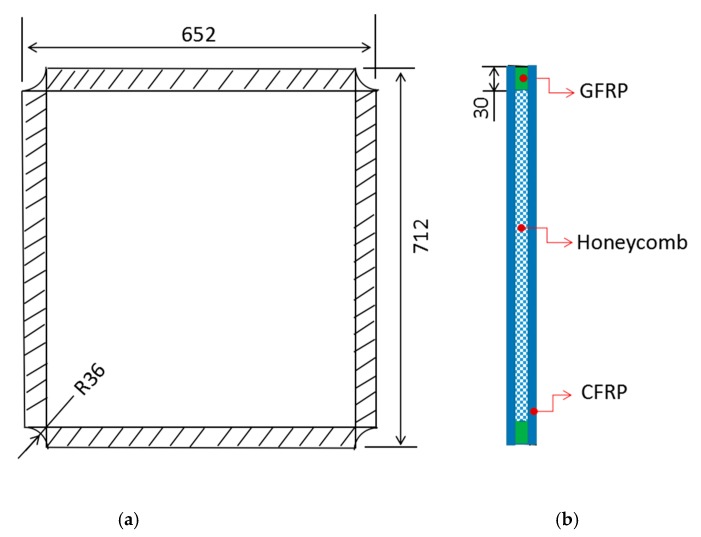
Schematic diagram of geometric dimension of the Nomex honeycomb sandwich CFRP panel: (**a**) top view of specimen; (**b**) side view of specimen (unit: mm).

In this study, the in-plane shear test pieces were designed according to true typical structure dimensions of a certain type of aircraft fuselage [21]. The honeycomb sandwich CFRP structures were laminated with an average thickness of 20 mm. The size of the shear specimen was 712 mm × 652 mm × 20 mm, and the effective testing range was 652 mm × 592 mm (r = 1.10). In order to avoid the destruction of the clamping area in advance, the honeycomb core in this area was replaced by a glass fiber reinforced plate. One quarter of a circle with a diameter of 36 mm was cut off from the four corners of the shear specimen to reduce the boundary stress concentration. The specimen geometry dimension is shown in Figure 2.

## 3. Experimental

### 3.1. Shear Test Fixture

**Figure 3:**
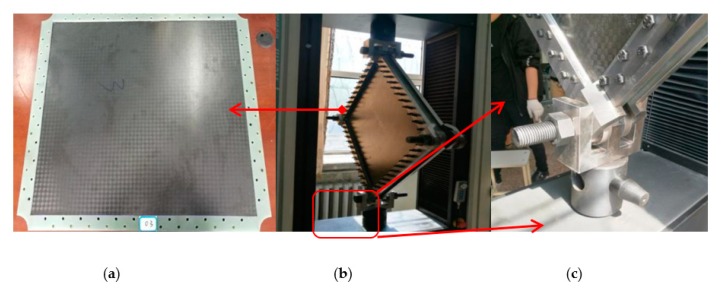
Shear test set-up: (**a**) specimen; (**b**) assembly component; (**c**) corner enlargement.

Based on the in-plane shear test method [30], a special picture frame type fixture was designed and fabricated in-house for applying shear loading on the honeycomb sandwich CFRP panel as shown in Figure 3. Typically, it is a hinged loading frame with eight arms of relative equal length bolted together using high strength steel bolts with diameter of 8 mm. Then 21 bolts were installed with a double row staggered arrangement on each side of the short side and 23 bolts were placed on each side of the long side. The fixture was designed in this way so that it enforces the clamped plate boundary conditions along the edges of the panel. Four corners of the picture frame were connected with bolts with a diameter of 40 mm which in turn allowed them to rotate freely upon loading. To ensure a proper clamping force, a consistent torque level of 25 Nm was applied to all the steel bolts using a rated torque wrench. The entire fixture assembly was mounted onto the loading frame as shown in Figure 3.

### 3.2. Test Equipment

Experimental investigations were conducted using an electronic universal material testing machine manufactured by Jinan Wanzhong Electrical Appliances Co., Ltd, Jinan, China, which has a load capacity of 300 kN and maximum stroke of 1500 mm. A vertical displacement-controlled loading was applied to the bottom end of the loading frame at a constant cross-head speed of 1 mm/min. The other end was fixed. The load applied on the specimen was measured by a 300 kN axial load cell. The displacement information was recorded at the crosshead by the test apparatus automatically. The crosshead moved up to a location till total failure of the specimen occurred.

**Figure 4:**
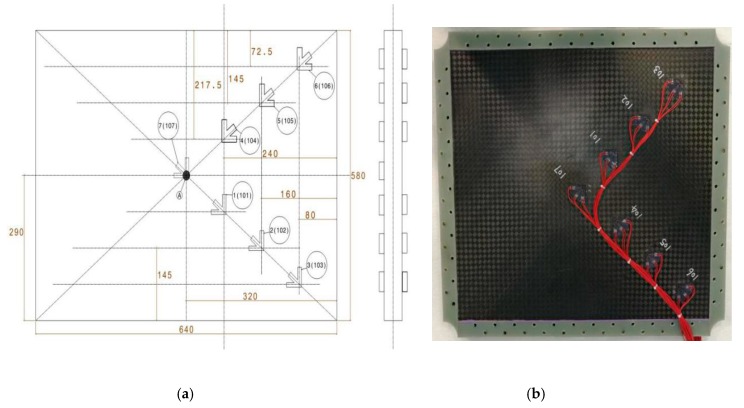
Position and number of strain gauge: (**a**) schematic diagram; (**b**) photo.

In order to monitor the shear strain field distribution and critical failure load of the test pieces, a set of strain gauges of 120 Ω capacity were used to measure the strain development in the test area on skin. All the strain gauges were symmetrically pasted on front and opposite sides. The number of the strain gauges on the opposite side is the number at the same position on the front side plus 100. The position and serial number of the strain gauges pasted are shown in Figure 4. The total number of the strain gauges was 14. The strain data were collected by JM5938 system provided by Yangjing corporation. 

The engineering shear strain was obtained by rosette strain gauges, whose number is the combination of j~j + 2. No. j measured the 0° direction strain along the length of specimen. No. j + 1 measured the 45° direction strain. No. j + 2 measured the 90° direction strain. The local shear strain measured by the rosette strain gauges was calculated as follows:

*Υ_xy_* = 2*ε*_45°_ − (*ε_x_* + *ε_y_*) (1)

where *Υ_xy_* is shear strain; *ε_x_* is 0° direction strain; *ε_y_* is 90° direction strain; and *ε*_45°_ is 45° direction strain.

In addition, random speckle patterns were made on the surfaces of the test panels for carrying out the whole field 3D-DIC measurement, a non-contact ARAMIS optical strain measurement system with a sampling frequency of 7Hz, which was used to verify the above strain acquisition, and to supplement the global strain field distribution. Three large size pieces subjected to in-plane shear loading were conducted on such a test system.

## 4. Theoretical analysis

### 4.1. Assumptions

**Figure 5:**
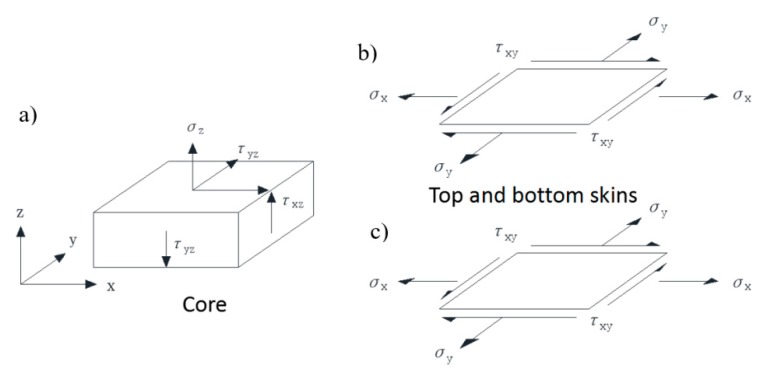
Stress diagram of honeycomb sandwich structure: (**a**) core; (**b**) top skin; (**c**) bottom skin.

Derivation of the shear strength distribution relationship of the honeycomb sandwich composite structure in the in-plane shear test is based on the following assumptions: (a) the normal tensile stiffness of the core is generally replaced by the compression stiffness because it is not easy to be measured; (b) the Poisson’s ratio effect and tension/compression/bending coupling are not considered; (c) the composite skin only bears the in-plane load. The main geometrical parameters of the honeycomb sandwich composite structure are shown in Figure 1. The stress state of each component of the sandwich structure is shown in Figure 5.

### 4.2. Equivalent Stiffness of Honeycomb Sandwich Composite Structure

From the assumptions above, the in-plane stiffness of the sandwich structure is mainly provided by the top and bottom skins. In the elastic stage, the lamina stiffness is given by the classical laminate theory [43]:

(2) $$Q_{11}=\frac{E_{1}}{1-\nu_{12}\nu_{21}},Q_{22}=\frac{E_{2}}{1-\nu_{12}\nu_{21}},Q_{12}=\frac{\nu_{21}E_{2}}{1-\nu_{12}\nu_{21}}=\frac{\nu_{12}E_{1}}{1-\nu_{12}\nu_{21}},Q_{66}=G_{12}$$

The stiffness relation between θ° and 0° lamina can be written as:

(3) $$\begin{array}{l} {\bar{Q}}_{11}=Q_{11}\cos^{4}\theta +2(Q_{12}+2Q_{66})\sin^{2}\theta \cos^{2}\theta +Q_{22}\sin^{4}\theta \\ {\bar{Q}}_{22}=Q_{11}\sin^{4}\theta +2(Q_{12}+2Q_{66})\sin^{2}\theta \cos^{2}\theta +Q_{22}\cos^{4}\theta \\ {\bar{Q}}_{12}=(Q_{11}+Q_{22}-4Q_{66})\sin^{2}\theta \cos^{2}\theta +Q_{12}(\sin^{4}\theta +\cos^{4}\theta )\\ {\bar{Q}}_{66}=(Q_{11}+Q_{22}-2Q_{12}-2Q_{66})\sin^{2}\theta \cos^{2}\theta +Q_{66}(\sin^{4}\theta +\cos^{4}\theta ) \end{array}$$

The stiffness of laminate can be calculated by the following equation:

(4) $$\begin{array}{l} A_{ij}=\sum_{1}^{n}(\bar{Q_{ij}}{)}_{k}(z_{k}^{}-z_{k-1}^{})\;i,j=1,2,6\\ D_{ij}=\frac{1}{n}\sum_{1}^{n}(\bar{Q_{ij}}{)}_{k}(z_{k}^{3}-z_{k-1}^{3})\;i,j=1,2,6 \end{array}$$

where *n* is the total number of layers; *k* denotes the *k*th layer; *A_ij_* is the tension stiffness; *D_ij_* is the bending stiffness; *z* is the distance from the center.

**Figure 6:**
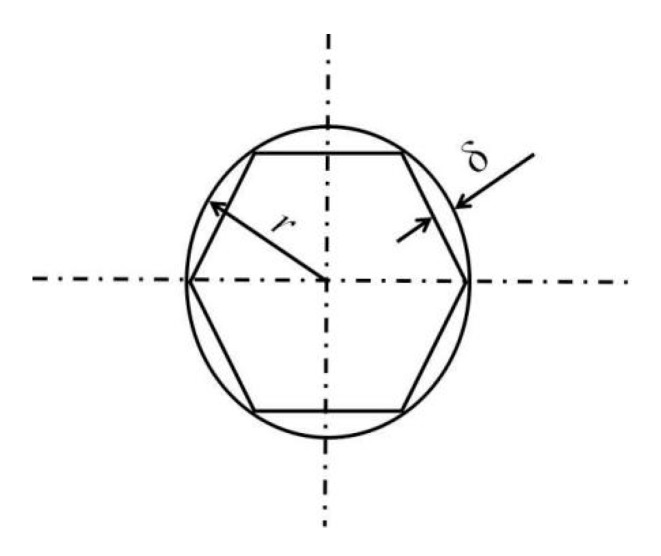
Diagram of honeycomb.

The stiffness of core is given as

(5) $$\begin{array}{l} {\bar{G}}_{xz}=0.8661\gamma \frac{G_{c}\delta }{r}\\ {\bar{G}}_{yz}=0.5774\gamma \frac{G_{c}\delta }{r} \end{array}$$

where *δ* is the thickness of the honeycomb thin wall, as shown in Figure 6; *r* is the circumferential radius of a regular hexagonal honeycomb hole; *γ* is a correction coefficient, which is a function of the parameters of manufacture, generally 0.4–0.6.

In case of the overall deformation, the sandwich panel similarly obeys the classical elasticity theory. Its neutral axis (see Figure 1) is solved by the following formula:

(6) $$Z_{g}=\frac{Ey_{i}\frac{e_{i}^{2}}{2}+Ey_{c}e_{c}\left(e_{i}+\frac{e_{c}}{2}\right)+Ey_{s}e_{s}\left(e_{i}+e_{c}+\frac{e_{s}}{2}\right)}{Ey_{i}e_{i}+Ey_{c}e_{c}+Ey_{s}e_{s}}$$

For a sandwich structure with thin skins, the bending stiffness can be simplified to

(7) $$Z_{g}=\frac{Ey_{i}\frac{e_{i}^{2}}{2}+Ey_{s}e_{s}\left(e_{i}+e_{c}+\frac{e_{s}}{2}\right)}{Ey_{i}e_{i}+Ey_{s}e_{s}}$$

where *s*, *i,* and *c* represent upper and lower panels and core respectively; and *e* represents thickness; *Ey* represents stiffness; *Ef* represents equivalent bending stiffness; *G* represents shear stiffness.

If the upper and lower skins are symmetric and have the same thickness, the functional expression of the bending stiffness coefficient along the x and y directions can be written as [44]

(8) $$D_{x}=\frac{E_{xs}e_{s}E_{xi}e_{i}t^{2}}{\left(E_{xs}e_{s}+E_{xi}e_{i}\right)\lambda }+\frac{1}{12\lambda }\left(E_{xs}e_{s}^{3}+E_{xi}e_{i}^{3}\right)$$

(9) $$D_{y}=\frac{E_{ys}e_{s}E_{yi}e_{i}t^{2}}{\left(E_{ys}e_{s}+E_{yi}e_{i}\right)\lambda }+\frac{1}{12\lambda }\left(E_{ys}e_{s}^{3}+E_{yi}e_{i}^{3}\right)$$

where *λ* = (1 − *ν*_xy_*ν*_yx_); *ν*_xy_ and *ν*_yx_ are the Possion’s ratio of lamina; *t* = *e_c_* + (*e_s_* + *e_i_*)/2.

The in-plane shear stiffness of the sandwich structure can be written as

(10) $$E_{e}=\frac{\sum_{k=1}^{3}G_{k}e_{k}}{\sum_{k=1}^{3}e_{k}}$$

(11) $$\frac{1}{G_{e}}=\frac{\sum_{k=1}^{3}\left(\frac{e_{k}}{G_{k}}\right)}{\sum_{k=1}^{3}e_{k}}$$

where *E_e_* and *G_e_* represent equivalent modulus of elasticity; *k* represents *s*, *i* or *c* (top skin, bottom skin or core).

**Figure 7:**
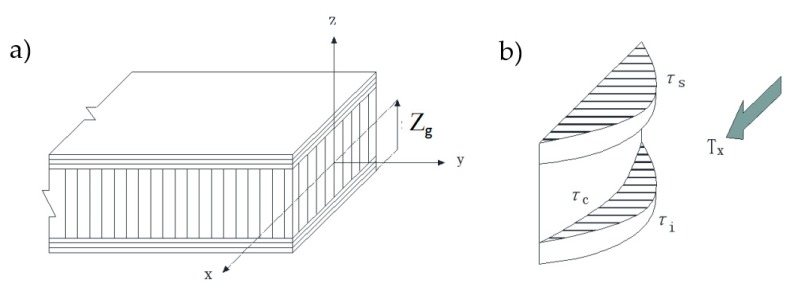
Stress distribution of sandwich structure under in-plane shear load: (**a**) coordinate system; (**b**) shear stress diagram.

To study the in-plane shear deformation characteristics of the sandwich structure, the shear load can be distributed to the top and bottom skin and core according to the shear stiffness ratio, as shown in Figure 7. The bearing shear stress of the skins and core can be calculated according to the following formula:

(12) $$\begin{array}{l} \tau_{s}=\frac{3}{2}\frac{T_{x}}{be_{s}}\frac{G_{s}e_{s}}{G_{s}e_{s}+G_{c}e_{c}+G_{i}e_{i}}\\ \tau_{c}=\frac{3}{2}\frac{T_{x}}{be_{c}}\frac{G_{c}e_{c}}{G_{s}e_{s}+G_{c}e_{c}+G_{i}e_{i}}\\ \tau_{i}=\frac{3}{2}\frac{T_{x}}{be_{i}}\frac{G_{i}e_{i}}{G_{s}e_{s}+G_{c}e_{c}+G_{i}e_{i}} \end{array}$$

### 4.3. Buckling of Honeycomb Sandwich Composite Structure

Once the shear stiffness and stress in the sandwich structure are known, the buckling is one of the major failure modes. For the symmetric and same thickness of lamination, it defined that f = 2e_s_, h = 2e_s_ + e_c_. When f/h < 0.03, wrinkle on the skin is the primary failure mode; when f/h falls into 0.03–0.04, the dominant role of overall buckling will depend on the crease formula used. Based on the Hoff and Mautner method, when f/h > 0.044, the overall instability is the main failure mode [45]. Here, the tested specimen is f/h = 0.0654, the expression of overall buckling under shear load is [46]:

(13) $$N_{xycrit}=\frac{\pi^{2}{\left(e_{c}+h\right)}^{2}E}{4b^{2}(1-\mu^{2})}K_{}$$

where *N_xycrit_* is the buckling shear force at unit width; *b* is the width of the plate; *K* is the buckling coefficient, which is a function of boundary conditions, stiffness, and buckling half wave number. *K = K_h_ + K_f_*.

**Figure 8:**
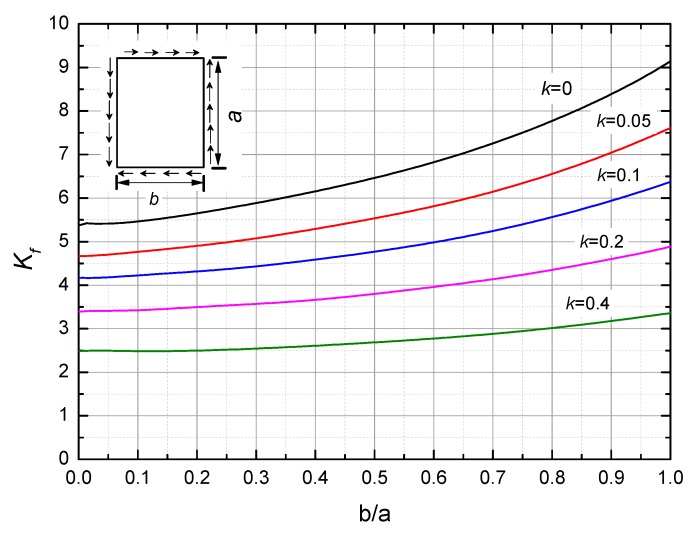
*K_f_* vs. *b/a* and *k* curves simply supported at four sides of the boundary condition.

*K_f_* was obtained by curve fitting with *b/a, k, g,* and the boundary condition, as show in Figure 8. *k* and *g* are given by

(14) $$\begin{array}{l} k=\frac{\pi^{2}hEe_{i}}{2\left(1-\mu^{2}\right)b^{2}{\bar{G}}_{xz}}\\ g=\frac{{\bar{G}}_{yz}}{{\bar{G}}_{xz}} \end{array}$$

The calculation formula *K_h_* is

(15) $$K_{h}=\frac{e_{i}{}^{2}K_{f0}}{3{\left(h+e_{i}\right)}^{2}}$$

where *K*_*f*0_ is the corresponding value of *K_f_* when *k* = 0.

## 5. Finite Element Model

**Figure 9:**
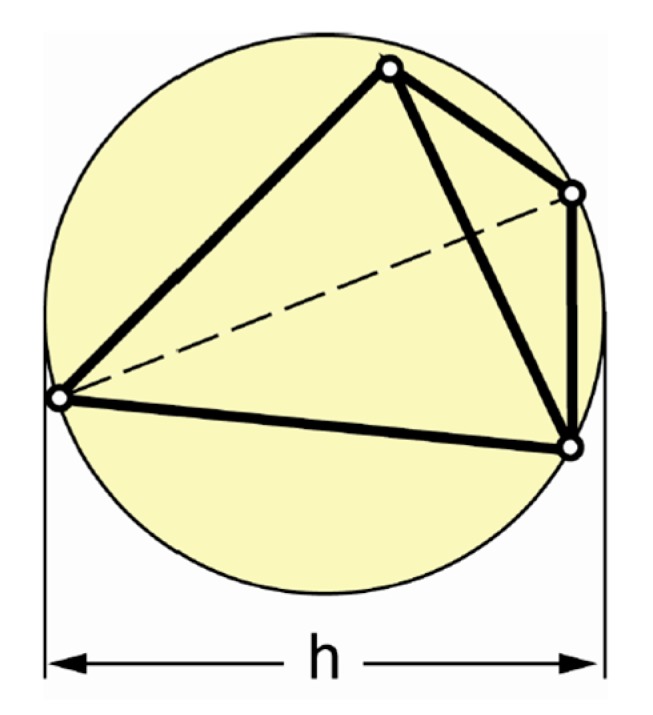
An element in a sphere solution domain.

The h element (characteristic element size) and p element (polynomial order element) were used to predict the shear field distribution and the overall instability load of the test piece, respectively. Figure 9 shows an element in a sphere. The h element is a traditional finite element method, which is an approximate method for solving differential equations cast in integral form. The h is the diameter of the smallest sphere that contains an element. It is proven that as h_maxғ_→0, the finite element solution converges to the exact solution. Further, p is the polynomial degree assigned to the element. In such cases of a 4-node tetrahedron, p = 1, and a 10-node tetrahedron p = 2, and so on. It was proven that when p_min_→∞, the finite element solution converges to the exact solution. Since h_max_→0 or p_min_→∞ cannot be realized, a reasonable h-mesh and a reasonable p-distribution need to be created. When the complexity of geometry forces the mesh generator to produce a very fine mesh, p = 3, 4, 5 is usually sufficient. The finite element (FE) software product based on the h-version used here is ABAQUS. The StressCheck is implementation of the p-version.

### 5.1. Mesh

**Figure 10:**
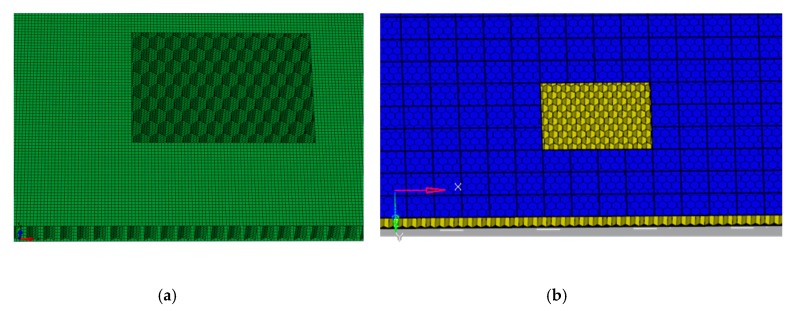
Schematic of the finite element model of honeycomb sandwich structure: (**a**) h element mesh; (**b**) p element mesh.

As shown in Figure 10, the h element of the in-plane shear test pieces was meshed to a size of about 5 mm, while the p element was meshed to 30 mm. The composite skins and core were defined as a kind of orthotropic shell. Both were meshed using reduced integration shell elements (S4R in ABAQUS). The polynomial degree of the p element model was set as p = 4 in StressCheck, ignoring the bonding surface between honeycomb and skins which was defined as a perfect contact. To ensure the capability of these models to represent the large size panel, a full-size model was created. The total number of h and p meshes was 109,004 and 28,007, respectively. The size of the fixture grids was about 30 mm, and the total number of grids was 380. The parameters of the Nomex material: *E*_1_ = 3500 MPa, *E*_2_ = 3200 MPa, *v*_12_ = 0.4, *G*_12_ = 1250 MPa, *G*_13_ = 1250 MPa, and *G*_23_ = 880 MPa. 

### 5.2. Boundary Conditions

**Figure 11:**
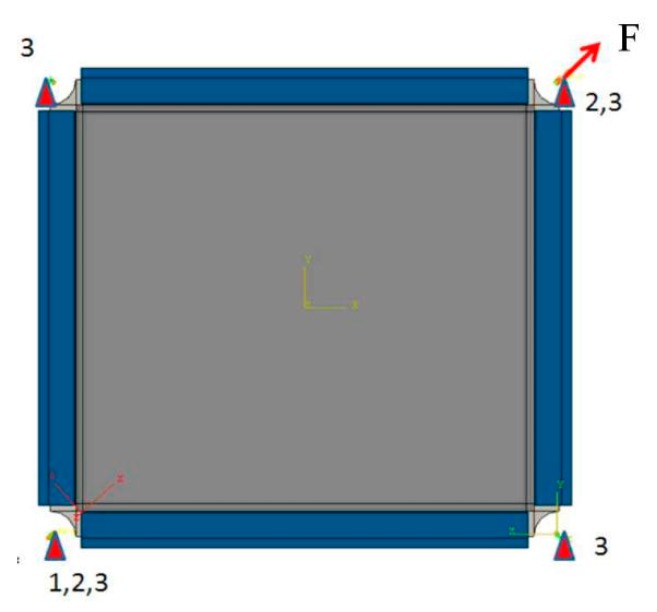
Boundary condition of in-plane shear test.

**Figure 12:**
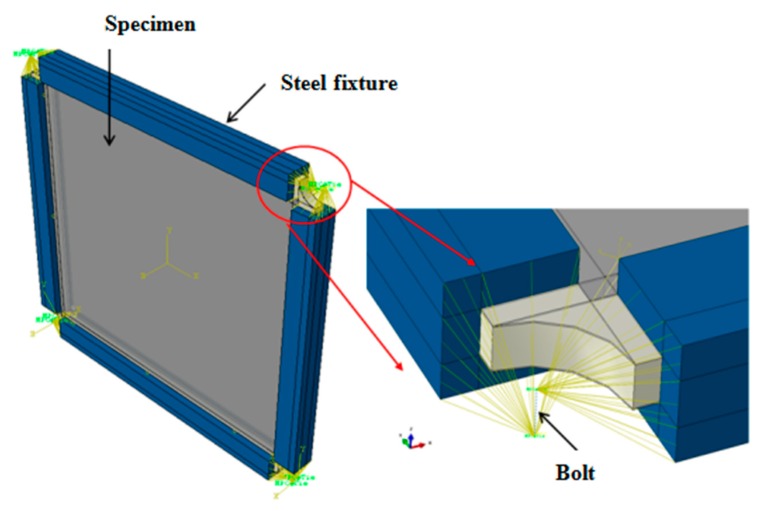
Schematic of the finite element model of shear test set-up.

As shown in Figure 11, for simulating the experimental boundary conditions, the panel dimension within the test fixture was considered. The panel edges were provided with a clamped boundary condition and were coupled to the reference points at the fixed and loading ends. Therefore, the clamped edges of the model act like rigid beams. The four corners of the model were bolted such that only rotation is allowed. CBUSH plus RBE3 bolted connection was used between the clamped edges of the fixture, as shown in Figure 12. The stiffness parameters of the CBUSH bolt are presented in Table 2. The load was applied at the bottom end of the panel which was allowed to move only in the loading direction, and the remaining degrees of freedom (DOFs) were fixed. 

### 5.3. Material Constitutive

A suitable material and constitutive model is fundamentally important to the validity of finite element prediction results. The constitutive and continuum damage models that are used to describe the deformation and damage response of the larger size sandwich structure consisting of lamina and the honeycomb are briefly introduced in this section.

Damage initiation was modeled using Hashin’s failure criteria [47] which includes four damage initiation mechanisms to detect respectively the failure modes in the matrix and fiber under both tension and compression failures. The failure modes included in Hashin’s criterion are as follows:

Mode I: fiber tension (${\hat{\sigma }}_{11}\geq 0$) 

(16) $$F_{f}^{t}={\left(\frac{{\hat{\sigma }}_{11}}{X^{T}}\right)}^{2}+\alpha {\left(\frac{{\hat{\tau }}_{12}}{S^{L}}\right)}^{2}\geq 1,where\leq \alpha \leq 10$$

Mode II: fiber compression (${\hat{\sigma }}_{11}\leq 0$)

(17) $$F_{f}^{c}={\left(\frac{{\hat{\sigma }}_{11}}{X^{c}}\right)}^{2}\geq 1$$

Mode III: matrix tension $\left({\hat{\sigma }}_{22}\geq 0\right)$

(18) $$F_{m}^{t}={\left(\frac{{\hat{\sigma }}_{22}}{Y^{T}}\right)}^{2}+{\left(\frac{{\hat{\tau }}_{12}}{S^{L}}\right)}^{2}\geq 1$$

Mode IV: matrix compression $\left({\hat{\sigma }}_{22}\leq 0\right)$

(19) $$F_{m}^{c}={\left(\frac{{\hat{\sigma }}_{22}}{2S^{T}}\right)}^{2}+\left[{\left(\frac{Y^{C}}{2S^{T}}\right)}^{2}-1\right]\frac{{\hat{\sigma }}_{22}}{Y^{C}}+{\left(\frac{{\hat{\tau }}_{12}}{S^{L}}\right)}^{2}\geq 1$$

where *F* is the determination value, the superscript *t* represents tensile; the superscript *c* represents compression; the subscript *f* represents fiber; the subscript *m* represents matrix; *X^T^* is the longitudinal tensile strength; *Y^C^* is the transverse compressive strength; *S^L^* is the longitudinal shear strength; *X^C^* is the longitudinal compressive strength; *Y^T^* is the transverse tension strength; *S^T^* is the transverse shear strength; α is a coefficient, which represents the contribution of the shear stress in model I.

The damage elastic matrix, which controls degradation of the material stiffness, can be expressed as:

(20) $$C_{d}=\frac{1}{D}\left[\begin{array}{ccc} \left(1-d_{f}\right)E_{1} & \left(1-d_{f}\right)\left(1-d_{m}\right)\nu_{21}E_{1} & 0\\ \left(1-d_{f}\right)\left(1-d_{m}\right)\nu_{12}E_{2} & \left(1-d_{m}\right)E_{2} & 0\\ 0 & 0 & \left(1-d_{s}\right)GD \end{array}\right]$$

where *G* is the shear modulus and *D* is an overall damage variable, which can be expressed as:

(21) $$D=1-\left(1-d_{f}\right)\left(1-d_{m}\right)\nu_{12}\nu_{21}$$

Here, *d_f_*, *d_m_*, and *d_s_* reflect the current state of fiber, matrix, and shear damage, respectively. *ν*_12_, *ν*_21_ are the Poisson’s ratios.

### 5.4. Buckling Load Analysis

ABAQUS provides two solvers to extract eigenvalues. In this paper, the mode superposition method of Subspace iteration solver was used to obtain the eigenvalues of linear buckling. StressCheck first solves the linear problem corresponding to the specified loads and constraints, then, utilizing the stress field computed from the linear solution, computes the geometric stiffness matrix, which is used for the eigenvalue computation. The first order positive eigenvalue (*λ_1st_*) of the sandwich structure was calculated, and then the total buckling load was obtained by multiplying the applied load (*P*_0_) and the B value reduction factor (0.9):

(22) $$P_{\mathrm{cr}}=0.9\lambda_{1\mathrm{st}}P_{0}$$

## 6. Results and Discussion

### 6.1. Load-Displacement Curves

**Figure 13:**
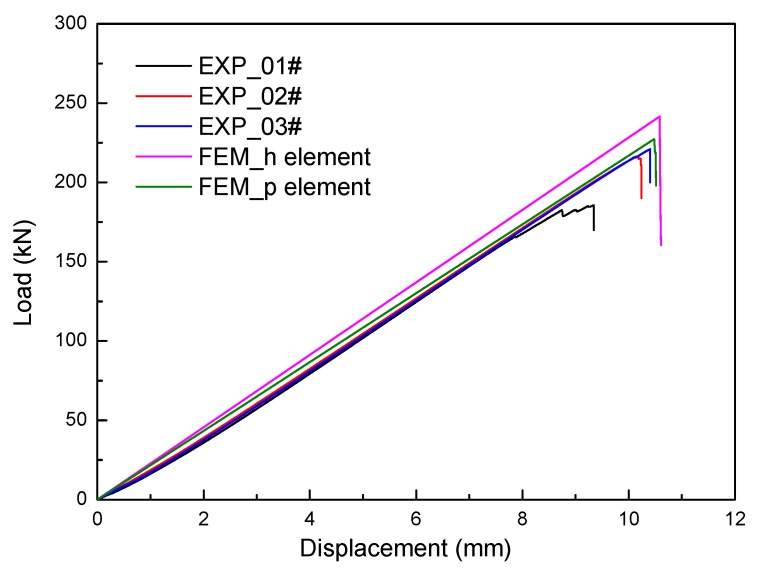
Shear load vs. displacement curves obtained by experimental and numerical methods.

**Figure 14:**
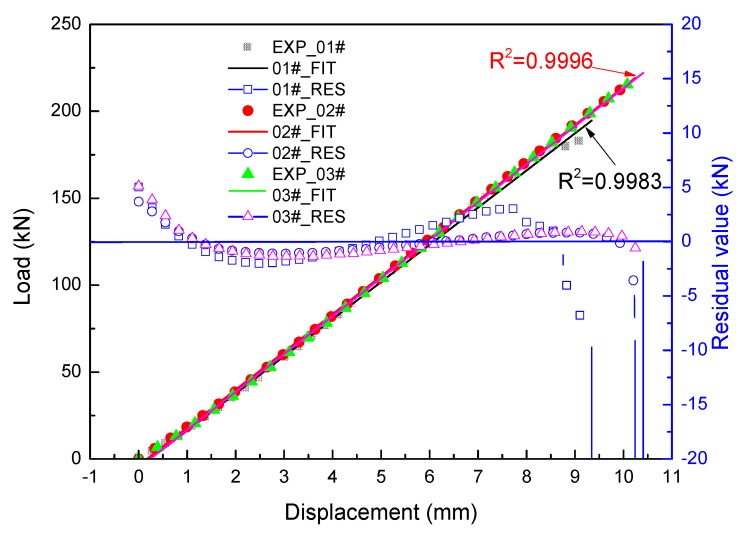
Load vs. displacement curves linear fitting and regular residual value curves.

Three specimens were loaded to catastrophic failure. The load vs. displacement curves of the sandwich structure are shown in Figure 13. It can be seen from the figure that the three experimental curves have good consistency with a low discrete coefficient of 2.3%, and the load response increases linearly with the increase of displacement until failure. The failure load is from 185 kN to 220 kN, and the average failure value is 205 kN. In case of 01# specimen, local damage occurs when the shear force reaches 181 kN. There are no other abnormal phenomena during the load process. The linearity of all three curves is greater than 0.99, as shown in Figure 14. The trend of the regular residual value between the test data and fitting curves shows that the structure is greatly affected by nonlinear factors, such as contact, gap, and geometry nonlinear on micro-scale, at the beginning and fracture stages. The discrete coefficient CV can be calculated by the following formula:

(23) $$CV=\frac{\sqrt{\frac{1}{N}\sum_{i=1}^{N}{\left(x_{i}-\mu \right)}^{2}}}{\mu }$$

where *x_i_* is the sample value, *N* is the sample size, and *μ* is the mean.

The linear correlation coefficient *R*^2^ can be calculated by the following equation:

(24) $$R^{2}=\frac{{\left(\sum_{i=1}^{n}\left(x_{i}-\bar{x}\right)\left(y_{i}-\bar{y}\right)\right)}^{2}}{\sum_{i=1}^{n}{\left(x_{i}-\bar{x}\right)}^{2}•\sum_{i=1}^{n}{\left(y_{i}-\bar{y}\right)}^{2}}$$

where *R*^2^ is the linear correlation coefficient, *x_i_* is x-value of the *i*_th_ array, $\bar{x}$ is abscissa mean of the entire array, *y_i_* is the y-value of the *i*_th_ array, $\bar{y}$ is the ordinate mean of the entire array.

From the discreteness and linearity analysis above, it can be concluded that the test data obtained from the three specimens have high reliability and good validity. Therefore, it is used to verify the feasibility of the developed FEM by comparing the displacement between FEM and experimental results. As presented in Figure 13, it could also be found that a generally acceptable accuracy is shown for the results predicted by the FEM and experimental results. The stiffness and failure load predicted by the h element model are greater than that of the p element model. 

### 6.2. Strain Analysis 

#### 6.2.1. Strain Data

**Figure 15:**
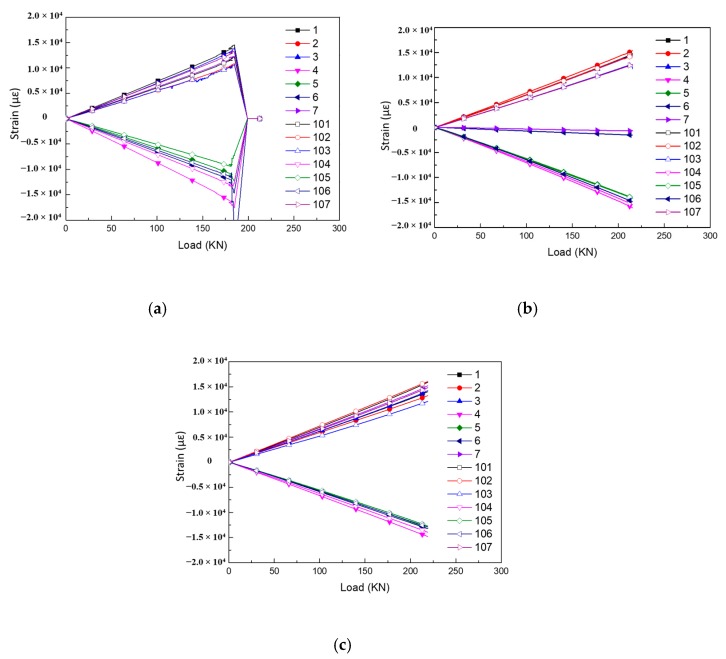
Shear strain vs. load curves of each specimen at the positions of strain gauge: (**a**) 01# specimen; (**b**) 02# specimen; (**c**) 03# specimen.

The strain data obtained from the gauges at the shear field of each test panel are shown in Figure 15. These curves show a good linearity response before shear failure, and the linearity is greater than 0.998. The orientations of strain gauge (SG) 4th–6th and 104th–106th on the top and bottom skins are rotated clockwise by 180° compared with 1st–3rd, which is not in accordance with the load direction. That leads to the shear strain values being opposite, but the absolute values of each shear strain are basically the same at the right position. It is obvious that the SG-6 and SG-7 on the 02# panel are invalid. The distribution of the shear strain is relatively uniform with good symmetry. The strain extremes corresponding to the failure load are listed in Table 3.

#### 6.2.2. Strain Discreteness

**Figure 16:**
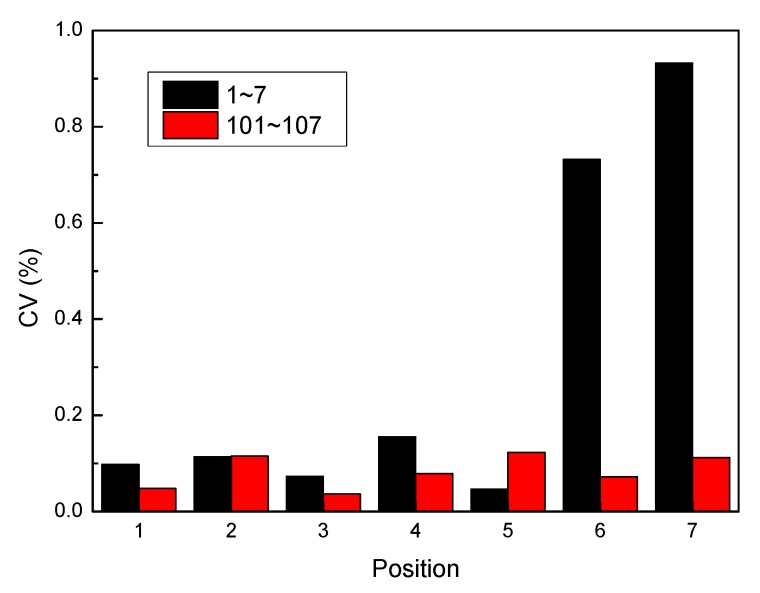
Discreteness of strain gauge data monitored under load of 180 kN.

In order to avoid accidental errors in the manufacture and single test load, the average value of the strain data of three specimens before failure (under 180 kN) was taken as the basis for analysis. Data exceeding 180 kN can be obtained by linear calculation, thus ensuring the reliability of the analysis results. The discreteness of the strain gauge data at the corresponding location is shown in Figure 16. It can be found that the most discrete coefficients are approximately within 15%, which confirm good repeatability of how the strain gauge data is obtained. Among them, the large discrete coefficients exceeding 15% were produced mainly because the 02# experiment produced invalid data, which will be deleted in the below analysis process.

#### 6.2.3. Comparison of Theoretical Prediction and Experimental Results

**Figure 17:**
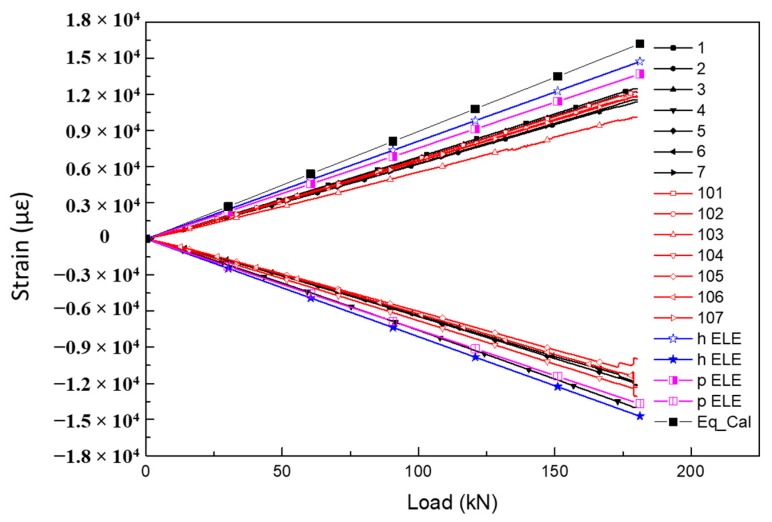
Comparison of strain vs. load curves at positions of strain gauge obtained from prediction and experimental.

The theoretical prediction contains theoretical analysis results calculated by a series of equations and two kinds of FEM models. Figure 17 shows the comparison of average shear strain vs. load curves obtained from the theoretical prediction and experiment under the shear load of 180 kN. Beyond that point, there is a marked deviation due to damage events occurring like delamination and fiber compression failure. It can be seen from Figure 17 that there is a good match with linear response among the three results. By comparing the maximum strain at the load of 180 kN, it is noticed that the theoretical predicted value is larger than the experimental value, which indicates the predicted value is conservative. The maximum strain is the theoretical equations calculation which makes several assumptions to the geometry and the mechanical parameters of the honeycomb sandwich composite structure. Similarly, with the displacement result, the p model result is closer to the experimental value than the h model. This is probably due to the p element increasing the polynomial order of the element without requiring mesh refinement. More features can be retained with less assumptions.

#### 6.2.4. DIC Strain Filed 

**Figure 18:**
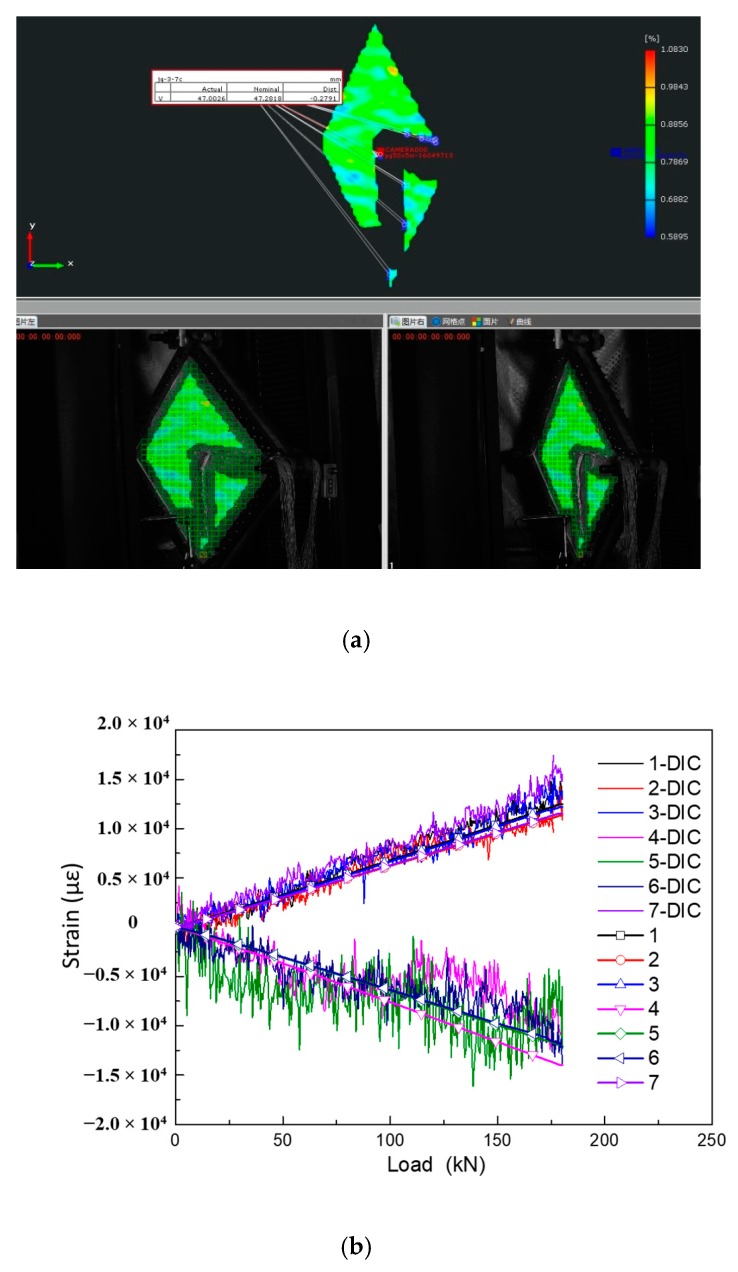
Comparison of DIC data and strain gauge data for shear test at the positive side: (**a**) DIC strain image; (**b**) shear strain curves.

The strain filed data measured by 3D-DIC is used to verify the above strain acquisition. Figure 18 shows the comparison of 3D-DIC shear strain data with the gauge monitor values and they are in close agreement. It conforms that the experimental results are effective and reliable. However, due to the size of the specimen, it is so large that it limits the assembly between the specimen and the testing machine, which results in that the camera of the testing system cannot be perpendicular to the surface of the test piece. There is an angle between the camera and the test piece, which makes the strain values in the two directions (direction a and c), which are 45° diagonal to the diagonal line, have a larger error. By comparing the two diagonal lines stain values with the direction b, the strain values of the two diagonal lines are an order of magnitude smaller than that of the direction b. It can be inferred that the principal strain direction of this kind of shear test is along the diagonal direction, which is in accordance with the test expectations. In addition, the DIC equipment adopts dynamic acquisition mode with a sampling frequency of 7 Hz, which is affected by clutter. The curves show a lot of jitters, while the trend is consistent with the gauge monitored.

### 6.3. Out-of-Plane Displacement Analysis 

**Figure 19:**
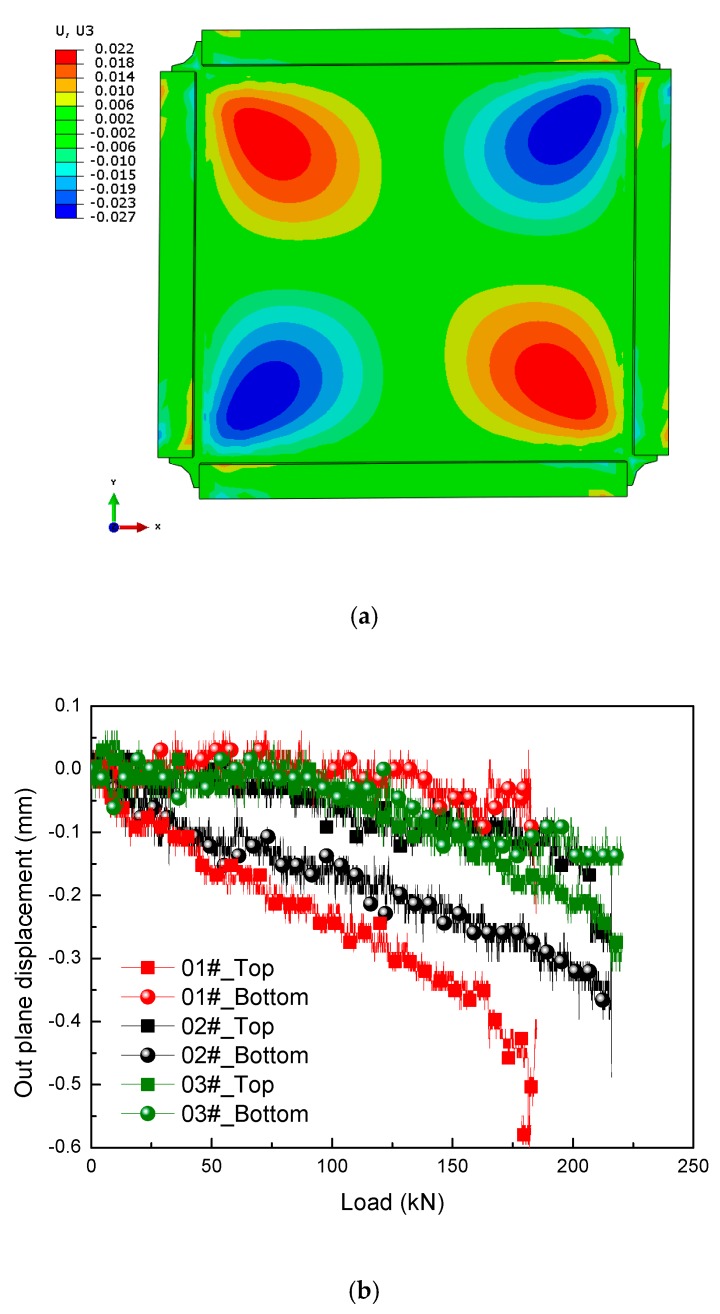
Out-of-plane displacement: (**a**) out-of-plane displacement field distribution obtained by finite element methods (FEM); (**b**) displacement vs. load curves recorded by experiment.

The out-of-plane displacement filed distribution under pure shear loading predicted firstly by the FEM method is shown in Figure 19a. It can be seen that there is an approximately symmetric deformation at the corners. The maximum deformation occurs near the place where No.2 (102) strain gauge is pasted (see Figure 4). Two deflection meters were used to measure the out-of-plane displacement of the sandwich panel in the area of No.2 (102) strain gauge symmetrically, and the sensor probes are placed vertically on the skin. The out-of-plane displacement vs. load curves recorded on both sides are shown in Figure 19. As can be seen from the figure, both out-of-plane displacements are negative, which means the panel is expanding due to Poisson’s ratio effect where it is subjected to tension load. Another interesting phenomenon is that the greater the out-of-displacement difference between the top and bottom skins, the smaller the bearing load could be captured among the test panels. This is because the ability to transfer shear force is strongest, when the top and bottom skins of the panel deform harmoniously. 

### 6.4. Strain Field Distribution 

**Figure 20:**
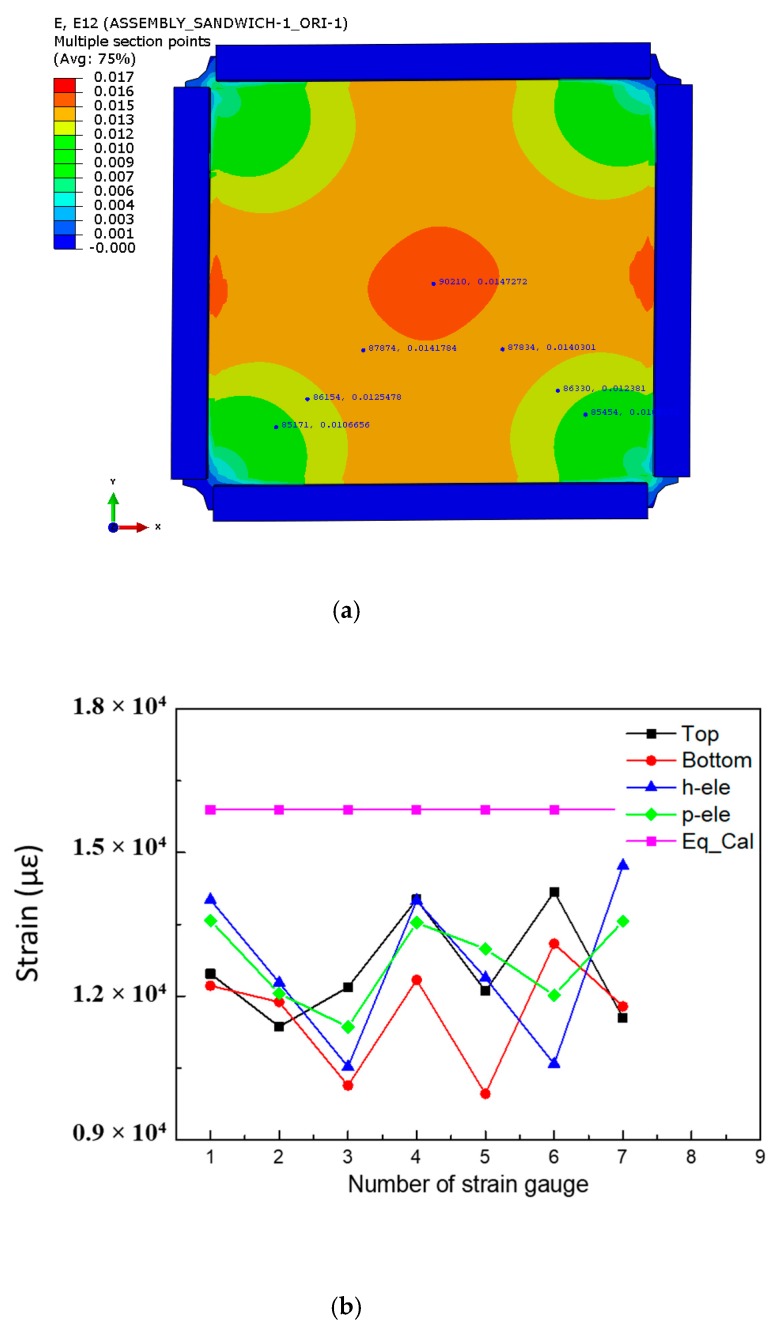
Strain field distribution: (**a**) predicted shear strain filed by FEM; (**b**) comparison of shear strain filed obtained by experimental and predicted methods.

To study the in-plane shear deformation characteristics of the large size honeycomb sandwich composite structure, the predicted field distribution of in-plane shear strain and the comparison of strain picked up at the No. 1–7 strain gauge positions under the load state of 180 kN are shown in Figure 20. It can be seen that a relatively uniform shear strain field is generated, and the strain gradually increases from the corners to the middle. The shear strains distribute approximately between 1000 με and 14,000 με. Comparing the results between the experimental method and numerical method, the distribution of strain field is similar. The deviation between the FEM predicted shear strain responses and the testing results was less than 10%, while the deviation between the theoretical predicted shear strain responses and the testing results was up to 15%. However, the theoretical analysis method is conservative, which meets the engineering requirements. 

### 6.5. Buckling and Failure Mode Characteristics 

**Figure 21:**
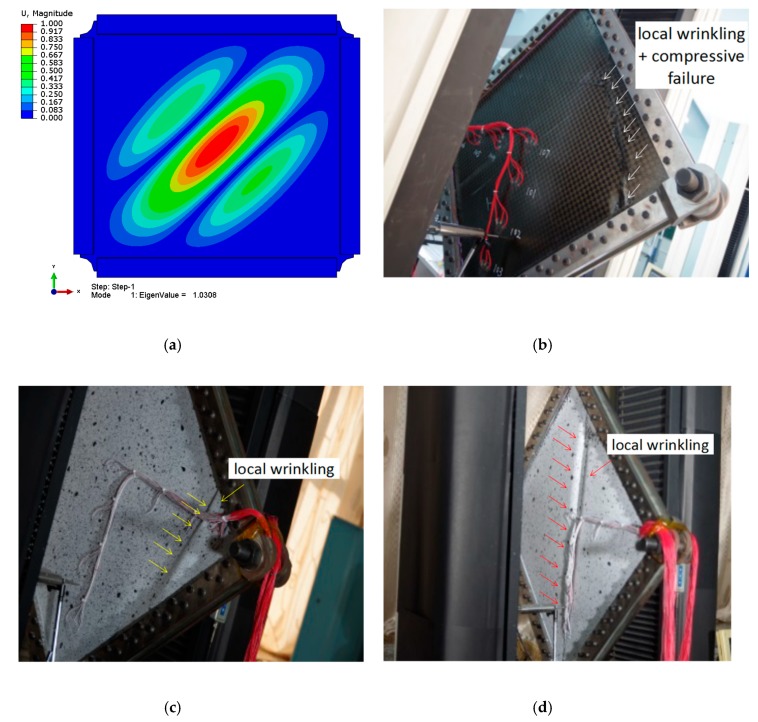
Buckling and failure mode: (**a**) first mode calculated by FEM; (**b**) failure modal of 01# sample; (**c**) failure modal of 02# sample; (**d**) failure modal of 03# sample.

From the displacement and strain analysis above, it can be concluded that the particular large size honeycomb sandwich composite structure has no post-buckling response. The whole shear buckling load of the specimen was predicted by FEM, as shown in Figure 21a. The first mode is 302 × 1.03 × 0.9 = 279.9 kN, and the first and second characteristic modes are very close. The buckling mode is the central buckling of the specimen (a half wave) under diagonal tension. However, due to the manufacture quality assurance problem for the such a large size sandwich composite, the effective overall buckling failure modes of the three specimens were not measured. Local wrinkling modes occur at the corner or diagonal tension direction, as shown in Figure 21b–d. This results in the failure load being relatively low, which could not be used to validate the buckling analysis methods. 

The overall buckling failure loads of the honeycomb sandwiched composite panel obtained by the above methods are summarized in Table 4. For the shear test, it can be concluded that the failure load calculated by the theoretical analysis method is smaller and more conservative than the FE predicted load. The experimental result is small because the local damage occurred before the overall instability. However, the deviation between FEM results and theoretical results is about 6.5%, which verifies the effectiveness of these two methods.

In summary, a comprehensive discussion on the in-plane shear response of the large-size honeycomb sandwich composites was given out. Firstly, we compared and analyzed the response of displacement based on the experimental recorded date and FEM predicted curves. Then we compared and discussed the experimental shear strain using the date of the strain gauge monitor system and DIC system. What is more, we verified and revealed the in-plane shear strain field distribution by FEM method and theoretical analysis method. In addition, we exhibited the distribution of the out-of-plane displacement in the FEM calculated field and provided and explained the relation between the experimental response of the out-of-plane displacement and the failure load of the honeycomb sandwich structure. Finally, we displayed the photos of the failure modes of the honeycomb sandwich structure along with a buckling analysis implemented by the FEM method and the theoretical analysis method. 

By comparing these methods, it was found that the strain gauge monitoring system is the most direct method to get responses, but the feedback information is limited. The DIC method can get a relatively complete information. However, it has to conduct a high cost test. The theoretical analysis method is simple and conservative, which means it is relatively inaccurate to predict the responses of the honeycomb sandwich composite structure. In addition, the information available is also limited. While the FEM method can obtain better prediction results when building a reasonable model, the p element and h element need to be selected reasonably according to the analysis situation.

## 7. Conclusions

In this work, a detailed experimental study on the in-plane shear response and failure mode of large size honeycomb sandwich composites with woven face sheets was conducted. A typical geometry and size of honeycomb sandwiched composite and a modified shear fixture were designed and manufactured. Three specimens were loaded to catastrophic failure in order to study the ultimate bearing capacity and failure modes. Two finite element methods, h-element and p-element, and theoretical analysis were employed to investigate the deformation characteristics, strain field distribution, and buckling mode under pure shear loading. The major conclusions are as follows:

- The experimental displacement and strain data show good repeatability and symmetry, which confirms the rationality of the experiment design for the large-scale sandwiched structure.
- The honeycomb sandwich composite shows linear response under in-plane shear load until final failure. The average failure load reaches 205.68 kN, and the maximum shear strain monitored on the composite plate is up to 16,115 με.
- Local wrinkling occurs at the corner or diagonal tension direction before overall buckling appears due to the manufacture quality assurance problem for the such large size honeycomb sandwich composite.
- The out-of-plane displacement filed distribution under pure shear loading had approximately symmetric deformation at the corners. The ability to transfer shear force for the sandwich structure mainly depends on the harmonious deformation between the top and bottom skins.
- A relatively uniform shear strain field was generated, in which the strain gradually increased from the corners to the middle.
- The results of the finite element method and theoretical analysis method were verified by comparing shear load vs. displacement curves, load vs. strain curves, and strain field distribution with experimental results.
- By comparing the experimental, FEM, and theoretical results, it can be concluded that the theoretical analysis method is relatively conservative, and the FEM method is more accurate in the case of deformation and strain. The results predicted by h element and p element methods are very close.
- The overall buckling loads obtained by the three methods are similar. Experiment results were relatively low because local failure occurs in advance. However, the FEM method and the theoretical analysis method can verify each other.

From all the above, it suggests the experimental method is flexible to evaluate the in-plane shear behavior of the large size specimen, the theoretical analysis method is relatively conservative in engineering analysis, the FEM methods are robustly applicable for the mechanical analysis of large scale structures. The results could provide data support for the comprehensive promotion of the design and application of honeycomb sandwich composites. 

## Figures and Tables

**Table 1:** Property parameters of carbon fiber reinforced polymer (CFRP) skin and Nomex core.

Property	CFRP Skin	Nomex Honeycomb Core
Longitudinal stiffness, *E_11_* (GPa)	36.65	0.015
Transverse stiffness, *E_22_* (GPa)	36.65	0.015
Out-of-plane stiffness, *E*_33_ (GPa)	10.16	0.175
Poisson’s ratio, *ν_11_*	0.04	0.4
Poisson’s ratio, *ν_13_*	0.31	0.1
Poisson’s ratio, *ν_23_*	0.31	0.1
Shear modulus, *G_12_* (GPa)	4.21	0.113
Shear modulus, *G_13_* (GPa)	4.23	0.077
Shear modulus, *G_23_* (GPa)	4.23	0.077
Longitudinal tensile strength, *X_t_* (MPa)	676	--
Longitudinal compressive strength, *X_c_* (MPa)	230	2.17
Transverse tensile strength, *Y_t_* (MPa)	676	--
Transverse compressive strength, Y_c_ (MPa)	230	--
Longitudinal shear strength, *S_12_* (MPa)	50	1.48
Transverse shear strength, *S_23_* (MPa)	75	0.84
Density, ρ (kg/m^3^)	1532	48

**Table 2:** Stiffness parameters of CBUSH bolt.

Axial Stiffness K_1_/(10^10^N·mm^−1^)	Shear Stiffness K_2_/(10^10^N·mm^−1^)	Shear Stiffness K_3_/(10^10^N·mm^−1^)	Rotational Stiffness K_4_/(10^10^N·rad^−1^)	Bending Stiffness K_5_/(10^10^N·rad^−1^)	Bending Stiffness K_6_/(10^10^N·rad^−1^)
1	1	1	0	1	1

**Table 3:** Shear strain extremes on composite skins corresponding to failure load.

Specimen	01#	02#	03#
Shear (με)	16,343	15,886	16,118
Failure load (kN)	185.56	210.63	220.84

**Table 4:** Buckling failure summary.

Method	Failure Load/N
Experiment	205,681
FEM_h ele	279,954
FEM_p ele	277,805
Theoretical analysis	260,433
